# BiGraph‐DTA: Predicting drug–target interactions of hepatoprotective agents with graph convolutional networks

**DOI:** 10.1002/qub2.70022

**Published:** 2025-11-12

**Authors:** Arief Sartono, Bambang Riyanto Trilaksono, Sophi Damayanti, Anto Satriyo Nugroho, Firdayani Firdayani

**Affiliations:** ^1^ School of Electrical Engineering and Informatics Institut Teknologi Bandung (ITB) Bandung Indonesia; ^2^ Research Center for Artificial Intelligence and Cyber Security Research Organization for Electronics and Informatics BRIN Bandung Indonesia; ^3^ School of Pharmacy Institut Teknologi Bandung (ITB) Bandung Indonesia; ^4^ Research Center for Vaccine and Drugs Research Organization for Health BRIN Bogor Indonesia

**Keywords:** BiGraph‐DTA, drug discovery, drug‐target affinity score prediction, graph convolutional network (GCN), hepatoprotective agents

## Abstract

Predicting drug–target affinity (DTA) is critical for discovering and developing hepatoprotective agents that can prevent and treat liver diseases. In this study, we propose BiGraph‐DTA, a new predictive model for identifying DTA score prediction for hepatoprotective compounds by combining graph convolutional networks and bidirectional long short‐term memory networks. This model is based on powerful frameworks that process both graph representations of molecular structures and sequential information from protein sequences to capture complex dependencies and interactions. Leveraging a curated hepatoprotective dataset (from ChEMBL) consisting of 21,421 interactions, the model outperforms traditional machine learning methods (such as random forest and XGBoost) as well as other deep learning methods (such as DeepDTA and GraphDTA) in terms of predictive performance. The BiGraph‐DTA obtained the best mean squared error of 0.7885, *R* ^2^ of 0.7208, and concordance index of 0.8508. Our proposed architecture holds potential for accelerating the drug discovery process of hepatoprotective therapy by highlighting the framework through which candidate drugs and their corresponding protein targets can be identified based on robust data‐driven knowledge. This model, therefore, provides a new opportunity for discovering new hepatoprotective compounds, which may also make it possible to speed up finding new liver disease drugs.

## 1 INTRODUCTION

The prediction of drug–target affinity (DTA) is a critical step in the development of new hepatoprotective agents designed to prevent or treat liver diseases. With the rapid evolution of machine learning (ML) and deep learning (DL), the field has seen significant progress, leading to more accurate and faster predictions of these interactions. DTA score prediction, on the other hand, provides a quantitative measure of the binding strength between a compound and its target protein, offering deeper insights beyond binary interaction outcomes. This enables the identification of potential lead compounds with higher efficacy, guides drug repurposing strategies, and accelerates the rational design of therapeutics by prioritizing candidates with the most promising pharmacological profiles. Hepatoprotective drugs safeguard the liver by mitigating inflammation, boosting antioxidant defenses, and promoting liver regeneration. Commonly used agents include silymarin, curcumin, and N‐acetylcysteine, and a comprehensive understanding of their pharmacological mechanisms is essential [1, 2]. Drug–target interactions are fundamental to the discovery of these agents [3, 4], and computational modeling and bioinformatics are increasingly used for virtual screening in the drug discovery process [5]. The integration of computer‐aided drug design, artificial intelligence (AI), and ML has improved the accuracy and efficiency of DTA models for liver diseases [3, 6]. In this context, graph neural networks (GNNs) show great promise for forecasting DTA, especially within complex biological networks like protein–protein interaction networks, thereby enhancing the precision of predicting how hepatoprotective drugs interact with biological pathways to combat liver damage [2, 7]. GNNs can also forecast docking scores and anticipate drug toxicity through quantitative structure–activity relationship modeling, which accelerates the discovery process and reduces the risk of adverse effects in clinical trials [8, 9, 10, 11].

Despite the promise of these approaches, they are often limited by several significant challenges. A major barrier to using GNNs for therapeutic advancement in liver diseases is the lack of reliable data on liver toxicity, which can lead to prediction errors and the selection of potentially hazardous medications. To address this, researchers must improve the quality and quantity of data used for algorithm training and employ robust cross‐validation techniques to ensure prediction reliability [12, 13, 14]. Furthermore, although models such as GraphDTA, which use graph convolutional networks (GCNs), have shown exceptional performance on large datasets such as Davis and KIBA [15, 16], they struggle with domain‐specific datasets for hepatoprotective agents because of a lack of sufficient structural information [17]. These models are also not equipped to handle the sequential data of protein amino acid sequences, which is vital for understanding drug–target interactions [18, 19]. Similarly, other models such as DeepDTA, which use 1D convolutional neural networks (CNNs) for both drug and protein data, are limited by their inability to capture long‐range dependencies within protein sequences [20]. Traditional ML algorithms, such as random forest and XGBoost, are even less suited for this task, as their linear nature and reliance on predefined features cannot effectively model the intricate non‐Euclidean relationships inherent in drug–target interactions [20, 21, 22].

To overcome these limitations, we introduce BiGraph‐DTA, a novel hybrid model that combines the strengths of GCNs with bidirectional long short‐term memory networks (BiLSTM). Our approach leverages GCNs to accurately model the structural dependencies of drug molecules, which previous research has shown to outperform CNNs in processing 2D SMILES data [20]. By integrating BiLSTM, a recurrent neural network adept at learning long‐range dependencies, our model can effectively process the sequential information of protein sequences—a critical component that GCN‐only models often overlook [23]. This hybrid architecture enables BiGraph‐DTA to capture both the structural and sequential aspects of drug–target interactions simultaneously, leading to more accurate predictions and improved generalization, even on smaller domain‐specific datasets related to hepatoprotective agents. This study’s goal is to create a GCN‐based DTA model that can be used to virtually test new hepatoprotective drugs by feeding the model chemical structures and predicting their interactions with key liver enzymes and receptors [24]. This research focuses on numerous significant liver proteins that serve as drug targets, including prostaglandin G/H synthase 2 [8, 25, 26], carnitine O‐palmitoyltransferase 1, cholesterol acyltransferase, fatty acid‐binding protein, glutaminase liver isoform, glycogen starch synthase, glycogen phosphorylase [27], and human hepcidin [28]. By advancing DTA prediction, BiGraph‐DTA offers a more robust and accurate solution for understanding the molecular interactions involved in liver protection and accelerating the development of new therapies.

## 2 RESULTS

### 2.1 Prediction performance

Results from our study of drug–target interactions prediction for hepatoprotective agents were particularly promising. The model showed high precision and recall, with a high mean squared error (MSE), an excellent *R* ^2^, and a large concordance index (CI) value. This implies that our system is precise in predicting drug interactions and liver disease pertinent targets. Most of the found candidate therapies are expected to cause important interactions with known targets of hepatoprotective mechanisms. These candidates are worthy of further experimental evaluation and development as therapeutic approaches for liver disease.

The research is consistent with the findings presented by Liu [29]. A robust framework for identifying hepatoprotective agents utilizing advanced in silico and in vitro methodologies, proposing a multitarget‐based polypharmacology prediction (mTPP) approach that amalgamates virtual screening with ML techniques to accurately predict compounds with substantial potential as hepatoprotective agents. Furthermore, according to Vernetti [30], the availability of multiscale experimental models, such as 3D microfluidic systems utilizing zebrafish and human cells, is a crucial element in enhancing the success rate of drug development.

To evaluate the effectiveness of our model, we compared the predicted interactions by our model with those predicted by other widely used methods for drug–target interaction prediction. Our GNN outperformed traditional ML methods (such as random forest or XGBoost) and DL architectures (such as CNNs). Our model to achieve this performance illustrates how powerful graph‐based representations of DTA are. The GCN approach was cautiously performed on the hepatoprotector dataset and exhibited the characteristic to accurately predict drug–target interactions for different diseases.

A novel GNN approach incorporating 3D structural information from protein–ligand binding poses has shown superior performance in virtual screening and pose prediction compared to docking and other DL methods [31]. The DeepNC framework, which leverages multiple GNN algorithms, improves the performance of DTA prediction on a benchmark dataset [32]. GNNs have been shown to be more effective in representing non‐Euclidean data such as drug‐like molecular structures and protein interaction networks, facilitating drug repositioning and accelerating drug discovery [7].

The ChemBL database was queried for pharmacological substances exhibiting interaction values with the target protein of the hepatoprotective drug. We gathered 21,421 interaction data points, comprising SMILES codes, protein sequences, and IC50 values, after the data preprocessing stages of the search results. Four hyperparameters were chosen for the GCN model: learning rate, optimizer, batch size, and epoch count. We conducted multiple trials with various combinations of learning rates and optimization techniques. The hyperparameter combination yielding the best average MSE, *R* ^2^, and CI values on the validation set was selected as optimal for modeling the test set. Initially, we conducted trials using hyperparameters chosen from extensive ranges, followed by model optimization.

We used learning rates [0.005, 0.0005, 0.00005] and then searched in tighter 3 regions around the best value of the parameter (e.g., referees indicated that the best value of the parameter is 0.0005). We tested the model using batch sizes of 32, 64, 128, and 256. We chose batch sizes spanning a reasonable range that would allow us to see the effects of small‐versus large‐batch performance. Smaller batch sizes, such as 32, were able to generalize better onto the validation set but required a greater number of iterations to reach convergence, whereas larger batch sizes (e.g., 256) converged quicker but sacrificed generalization quality. Afterward, we batched sizes based on the above results and eventually, we found that 64 was the best parameter. Among all the selected new GNN configurations, the new batch size gave the best compromise between training time and performance on the validation dataset.

From Table 1, for strong optimizers, we also compared how Adam, RMSProp, SGD, and so on worked. Each optimizer was evaluated by its potential to maximize the drop of both training and validation loss and increase MSE, *R* ^2^, and CI. Adam was first applied broadly as an efficient optimizer because of its use of an adaptive learning rate appropriate for very sparse gradients. We trained on [100, 500, and 1000] epochs to study the best epochs in testing. After each epochs, we separated loss in validation and loss in training. At around 1000 epoch, the validation loss had flatlined, meaning more training will not result in lower value.

**TABLE 1:** Best hyperparameter for the graph convolutional network model.

No	Hyperparameter	Setting
1	Learning rate	0.0005
2	Batch size	64
3	Optimizer	RMSProp
4	Epoch	1000

Table 2 shows that different models outperform other state‐of‐the‐art methods capable of predicting protein–ligand binding affinity on comparative results. A variety of key indicators corroborate this assumption. The MSE of GCN is 0.8059, which is better than those of DeepDTA, random forest, and XGBoost. A low MSE signifies that the model succeeds at minimizing the difference between expected affinity values and real affinity values. Note that the three evaluation metrics shown for each row are the test results with the best iteration obtained from the 1000 allocated epochs. This context also applies for the remaining tables in this paper.

**TABLE 2:** Prediction performance on the hepatoprotector dataset.

Method	Protein rep.	Compound rep.	Best MSE	Best *R* ^2^	Best CI
Davis dataset [15]					
GCN	1D	Graph	0.2540	0.6931	0.8800
KIBA dataset [15]					
GCN	1D	Graph	0.1390	0.7044	0.8890
Hepatoprotector dataset					
**GCN**	1D	Graph	**0.8059**	**0.7167**	**0.8423**
**GCN + SVR**	1D	Graph	1.9373	0.3189	0.7016
**GCN + RF regressor**	1D	Graph	1.0725	0.6230	0.8083
**GCN + XGBoost**	1D	Graph	1.1224	0.6054	0.7990
DeepDTA	1D	1D	1.1355	0.6008	0.6008
Random forest	1D	1D	1.3490	0.3210	0.7200
XGBoost	1D	1D	1.1270	0.4250	0.7610

Abbreviations: GCN, graph convolutional network; MSE, mean squared error.

*Note*: The results of the experiment with the hepatoprotector dataset and the GCN method outperformed other experiments with the same dataset. Meanwhile, the methods in the first column that typed in bold font indicate (or emphasize) that the method uses a graph‐based algorithm.

The GCN *R* ^2^ score estimate was 0.7167, indicating that it explains 71.67% of the variance within our sample. If there is enough data in the input data itself (chemical structures or protein sequences), it shows its ability to accurately capture the complex correlation between target prediction and the input data. In the model, its CI of 0.8423 further confirmed its excellent performance by employing a statistically sound methodology to accurately rank binding affinity predictions.

Interestingly, when stacking GCN with popular ML regressors (i.e., SVR, RF, and XGBoost), the worst predictability was obtained. Specifically, GCN + SVR achieves the lowest MSE (1.9373) and the lowest *R* ^2^ (0.3189), both of which illustrate its poor predictive power. Also, GCN + RF and GCN + XGBoost improved slightly over SVR, but their performance is still below the independent GCN. However, in comparison, simpler models, such as random forest and XGBoost, underperform by predicting more errors and a lower predictive score. Results highlight that graph‐based representation learning methods, particularly GCN, were more appropriate for the prediction of hepatoprotectors compared to other ML methods, as indicated by their better predictive performance in the conducted experiments.

After performing hyperparameter tuning on the GCN model, it proved to achieve the best performance with the lowest MSE of 0.8059, the highest *R* ^2^ of 0.7167, and the highest CI of 0.8423. For instance, GCN performed significantly better than DeepDTA (MSE = 1.1355, *R* ^2^ = 0.6008) and random forest (MSE = 1.3490, *R* ^2^ = 0.3210) in predicting binding affinity since they averaged over models. Though random forest was outperformed by decision tree‐based models such as the XGBoost (MSE = 1.1270, *R* ^2^ = 0.4250), XGBoost, in turn, was outperformed by GCN.

We note that the poor performance of the GCN model on the hepatoprotector dataset relative to the much larger KIBA and Davis datasets. For the KIBA and Davis datasets, the GCN resulted in an MSE of 0.1390 and a CI of 0.8890, and a MSE of 0.2540 and a CI of 0.8800, respectively [33]. The vast scale and variety of these datasets are the main reasons for that gap in accuracy. Though KIBA and Davis have about 100,000 samples more than hepatoprotector, where there were only about 21,421 rows, which means limiting the training data for the model.

The first experiment was conducted using random forest and XGBoost directly from the data—in this case, the SMILES code and protein sequence only needed to be turned into sequence format and did not require further extraction of information. The results summed up below show that random forest had an MSE of 1.469059, an *R* ^2^ of 0.483536, and a CI of 0.759174. This large MSE value is a sign that this model is not well suited to predict accurate affinity. The comparison results of MSE, *R* ^2^, and CI for other models in contrast with XGBoost are depicted in Table 2. XGBoost and random forest are both model ensembles based on a decision tree. However, their fundamental mechanisms are distinct. The random forest algorithm functions by operating on each tree independently. Conversely, the XGBoost algorithm operates sequentially, wherein each tree is responsible for correcting the mistakes made by the preceding tree. The boosting approach, which systematically optimizes the loss function, frequently enables XGBoost to identify more complex data patterns and achieve superior performance, as evidenced in this analysis.

The second experiment leveraged a GCN to extract features from SMILES code and protein sequence before deploying them to additional regression models. This led to a MSE of 1.9373, *R* ^2^ of 0.3189, and CI of 0.7016 when using GCN input and SVR. The results suggest that the coupling of GCN and SVR is not responsive for this task, probably because of the lower fitting capability of SVR by the detailed features derived from GCN. As seen in Table 2, the GCN + RF regressor consistently outperformed a single GCN, with MSE of 1.0725, *R* ^2^ of 0.6230, and CI of 0.8083, which means that random forest is more capable of using the features extracted from GCN. In addition, combination GCN and XGBoost achieved an MSE of 1.1224, *R* ^2^ of 0.6054, and CI of 0.7990. Although GCN + XGBoost achieves better performance than XGBoost without GCN, it shows little inferiority against GCN + RF regressor.

### 2.2 Transfer learning

In this experiment, the transfer learning method was employed to evaluate and comparatively analyze previously applied models. The results of this evaluation are displayed in the following Table 3.

**TABLE 3:** Transfer learning using the KIBA dataset.

Epoch	Train loss	Val loss	Best MSE	Best *R* ^2^	Best CI	Description
1000	**0.1021**	**0.8509**	**0.7964**	**0.7200**	**0.8417**	Unfreeze the previous layer
1000	2.5248	2.4884	2.4807	0.1279	0.6333	Freeze all layers except the last layer
1000	2.2635	2.2290	2.0730	0.2712	0.6836	Unfreeze the previous layer, added some training layers
1000	1.5217	1.4381	1.3209	0.5356	0.7798	Unfreeze layer fc1, fc2, and out
1000	0.1889	0.9223	0.8846	0.6890	0.8328	Unfreeze last layer gcn fc_g2, prot seq fc1_xt, fc2, and out

Abbreviation: MSE, mean squared error.

*Note*: The results of the first experiment within the first scenario outperformed the experiments with the other scenarios. Each scenario details are described in the description column. The train loss, val loss, and best CI values in the first experiment should be bold as well, considering these values also outperformed the other experiments values.

In Table 3, without a freezing layer in transfer learning, the model achieves 0.7964 MSE, so it is just slightly better than GCN (0.8059) after fine‐tuning hyperparameters. It had a slightly better *R* ^2^ (0.7200 vs. 0.7167), but the CI was about the same. During training, we observed that the training loss increased to 0.1021, and there was a significant drop in performance when all layers were frozen except for the last one. This degradation led to a dramatic falloff in physical performance metrics, with MSE hitting 2.4807, *R* ^2^ coming down to 0.1279, and CI dropping to 0.6333. This result shows the KIBA dataset still lacks the adaptability required for immediate use on the target dataset without some degree of fine‐tuning.

Adding more layers to the model before unfreezing the previous layers decreased the performance metrics. The model demonstrated poor performance, yielding a MSE of 2.0730 and an *R* ^2^ value of 0.2712. Both metrics indicate that the model’s predictive accuracy is below acceptable limits. This approach indicates that the added restrictions limit the model’s transfer to the dataset used. With an unfreeze strategy, freezing all convolutional layers and enabling fine‐tuning but only on the FC layers (i.e., fc1, fc2, and out), performance improved slightly (at least in terms of MSE, we see 1.3209 as compared to 2.244) with *R* ^2^ = 0.5356. But this approach still scored significantly worse than the GCN model even without transfer learning. Under transfer learning with KIBA, the best results were obtained with the unfreezing of multiple GCN layers (fcn_g2), protein sequence (fc1_xt) and the core FC layers (fcn2, out) as well. This result is also supported by an MSE of 0.8846, *R* ^2^ of 0.6890, and a CI of 0.8328. However, the performance of the approach is still weaker as compared to the GCN approach with hyperparameter tuning.

When the Davis dataset was used in transfer learning without a freezing layer in Table 4, an MSE of 0.8129, an *R* ^2^ of 0.7142, and a CI of 0.8466 were achieved. The MSE was slightly lower in GCN compared to the other after being tuned, while the *R* ^2^ and the CI saw moderate improvements, indicating that the Davis dataset is a reasonable candidate for transfer learning. However, the performance dropped sharply with an unfrozen final layer only—with an MSE = 2.3257, an *R* ^2^ = 0.1824, and CI = 0.6708. Following this line of reasoning, the resulting performance mirrors what was observed from the transfer learning using KIBA; excessive freezing results in the model not being able to generalize well toward the target dataset.

**TABLE 4:** Transfer learning using the Davis dataset.

Epoch	Train loss	Val loss	Best MSE	Best *R* ^2^	Best CI	Description
1000	0.0878	**0.8611**	**0.8129**	**0.7142**	**0.8466**	Unfreeze the previous layer
1000	2.5746	3.5403	2.3257	0.1824	0.6708	Freeze all layers except the last layer
1000	**0.0863**	0.9895	0.8248	0.7100	0.8459	Unfreeze the previous layer, added some training layers
1000	0.9057	1.1802	1.1123	0.6090	0.8032	Unfreeze layer fc1, fc2, and out
1000	0.2127	0.9988	0.9451	0.6678	0.8280	Unfreeze last layer gcn fc_g2, prot seq fc1_xt, fc2, and out

Abbreviation: MSE, mean squared error.

*Note*: The results of the experiment within the first scenario (except for train loss value) outperformed the experiments with the other scenarios. Each scenario details are described in the description column. The val loss, best MSE, and best CI values in the first experiment should be bold as well, considering these values also outperformed the other experiments values. As for the best train loss value, is in another method (in this table, the third row).

In cases where added layers are frozen, the model returns an MSE of 0.8248 and *R* ^2^ of 0.7100. This outperforms GCN after tuning, but this is worse than freezing all layers. We unfroze only the FC layers and (fc1, fc2 and out), which resulted in MSE = 1.1123, *R* ^2^ = 0.6090, still a way to go to reach the best performance seen. In contrast, the other case of having unfreeze for multiple layers GCN (fc_g2), protein sequence (fc1_xt), and top FC layer (fc2, out) gave an MSE of 0.9451, *R* ^2^ of 0.6678, and a CI of 0.8280. These metrics do not reach the performance of the GCN after hyperparameter tuning.

### 2.3 Regularization

Experiments with Lasso (L1) in Table 5 regularization demonstrated that the application of regularization to all layers resulted in extreme underfitting of the model. This was evidenced by a train loss of 25,563,558.8828 and a val loss of 79,493.4170, as well as significantly worse performance metrics in comparison to the baseline. This finding indicates that the application of the L1 penalty to all model parameters results in the removal of a substantial number of important features.

**TABLE 5:** Regularization using Lasso.

Parameter	Epoch	Train loss	Val loss	Best MSE	Best *R* ^2^	Best CI	Description
L1 = 0.00001, batch size = 64, LR = 0.00005, optimizer = RMSprop	1000	25,563,558.8828	79,493.4170	79,493.4766	−27945.8330	0.5000	All layer
L1 = 0.00001, batch size = 64, LR = 0.00005, optimizer = RMSprop	1000	80.7197	0.9348	0.9342	0.6716	0.8387	Layers fc1, and fc2 (fully connected layers after concatenation)
L1 = 0.00001, batch size = 64, LR = 0.00005, optimizer = RMSprop	1000	**73.7446**	0.9071	0.9068	0.6812	**0.8390**	Layer fc_g1, fc_g2, fc1_xt (fully connected layers on smile block and protein block)
L1 = 0.00001, batch size = 64, LR = 0.00005, optimizer = RMSprop	1000	94.9761	**0.8954**	**0.8946**	**0.6855**	0.8384	Layer fc1, fc2, fc_g1, fc_g2, fc1_xt (fully connected layer after concatenation, and on smiles block and protein block)

Abbreviation: MSE, mean squared error.

*Note*: The best values for each metric are spread across the last two scenarios. Each scenario details are described in the description column.

However, when L1 regularization is applied to specific layers in Table 5, the model demonstrates enhanced performance. Specifically, in layers fc1 and fc2 (fully connected [FC] after concatenation), validation loss reduces to 0.9348, accompanied by metrics such as MSE = 0.9342, *R* ^2^ = 0.6716, and CI = 0.8387. In layers fc_g1, fc_g2, and fc1_xt (FC in SMILES and protein blocks), slightly improved results were observed, with MSE = 0.9068, *R* ^2^ = 0.6812, and CI = 0.8390.

Applying regularization to all FC layers (fc1, fc2, fc_g1, fc_g2, and fc1_xt) results in a negligible performance drop: MSE rises to 0.8946, *R* ^2^ increases to 0.6855, and CI increases to 0.8384. Cross‐expert sparsification on the FC layers after concatenation (fc1 and fc2) is a powerful method for significantly improving model performance with a negligible learning slowdown.

Experiments with Ridge (L2) in accordance with Table 6 demonstrate a trend similar to the previous one with its own particularities. We observe that the model was very error‐prone (train_loss = 1.539 × 10^19^) across the training viewport and proceeded to perform poorly (val_loss = 2.886, *R* ^2^ = −0.0146, CI = 0.5000) when regularization was applied on all layers. This indicates numerical instability because of an over‐imposing L2 penalty. On the other hand, with L2 applied only to layers fc1 and fc2, the model achieved MSE = 0.9079, *R* ^2^ = 0.6808, CI = 0.8424, a modest improvement over Lasso. The performance is almost comparable under this layer for fc_g1, fc_g2, and fc1_xt, for MSE = 0.9086, *R* ^2^ = 0.6806, CI = 0.8390, suggesting that a steady state of regulatory effects is in effect. As such, when all FC layers were applied, model performance diminished (MSE = 1.0073, *R* ^2^ = 0.6459, CI = 0.8320), implying that excessive regularization may limit the model’s performance. Again, the best Ridge regularization was applied only to the same layers as with Lasso results, but with better stability.

**TABLE 6:** Regularization using Ridge.

Parameter	Epoch	Train loss	Val loss	Best MSE	Best *R* ^2^	Best CI	Description
L2 = 0.00001, batch size = 64, LR = 0.00005, optimizer = RMSprop	1000	15,391,920,340,724,672,512.0000	2.8861	2.8860	−0.0146	0.5000	All layer
L2 = 0.00001, batch size = 64, LR = 0.00005, optimizer = RMSprop	1000	**43.4002**	**0.9081**	**0.9079**	**0.6808**	**0.8424**	Layers fc1, and fc2 (fully connected layers after concatenation)
L2 = 0.00001, batch size = 64, LR = 0.00005, optimizer = RMSprop	1000	45.5513	0.9090	0.9086	0.6806	0.8390	Layer fc_g1, fc_g2, fc1_xt (fully connected layers on smile block and protein block)
L2 = 0.00001, batch size = 64, LR = 0.00005, optimizer = RMSprop	1000	47.7102	1.0075	1.0073	0.6459	0.8320	Layer fc1, fc2, fc_g1, fc_g2, fc1_xt (fully connected layer after concatenation, and on smiles block and protein block)

Abbreviation: MSE, mean squared error.

*Note*: The results of the experiment within the second scenario outperformed the experiments with the other scenarios. Each scenario details are described in the description column.

ElasticNet uses the Lasso and Ridge methods together in Table 7, providing more stable results than if Lasso or Ridge were used independently. Regularization through all layers led to an MSE of 0.9541, an *R* ^2^ of 0.6646, and a CI of 0.8282, significantly better than Ridge and slightly worse than Lasso. When all layers are removed apart from fc1 and fc2, the performance is far better, which gives MSE of 0.8992, *R* ^2^ of 0.6839, and CI of 0.8381. When L1 and L2 were applied to layers fc_g1, fc_g2, and fc1_xt, performance was 0.0626 worse than the performance of Lasso or Ridge, respectively (MSE = 0.8888, *R* ^2^ = 0.6875, CI = 0.8431), but remained competitive. For the case when it is applied to all the connected layers, the performance values are pretty good, yielding MSE = 0.9028, *R* ^2^ = 0.6826, and CI = 0.8393. Nevertheless, it performs less well than applying only to fc1 and fc2. Overall, regularizing just fc1 and fc2 in accordance with ElasticNet achieves the best performance, retaining a better trade‐off compared to either Lasso or Ridge alone.

**TABLE 7:** Regularization using ElasticNET.

Parameter	Epoch	Train loss	Val loss	Best MSE	Best *R* ^2^	Best CI	Description
L1 = 0.00001, L2 = 0.00001, batch size = 64, LR = 0.00005, optimizer = RMSprop	1000	145.6873	0.9549	0.9541	0.6646	0.8282	All layer
L1 = 0.00001, L2 = 0.00001, batch size = 64, LR = 0.00005, optimizer = RMSprop	1000	**46.0860**	0.8999	0.8992	0.6839	0.8381	Layers fc1, and fc2 (fully connected layers after concatenation)
L1 = 0.00001, L2 = 0.00001, batch size = 64, LR = 0.00005, optimizer = RMSprop	1000	60.2217	**0.8892**	**0.8888**	**0.6875**	**0.8431**	Layer fc_g1, fc_g2, fc1_xt (fully connected layers on smile block and protein block)
L1 = 0.00001, L2 = 0.00001, batch size = 64, LR = 0.00005, optimizer = RMSprop	1000	79.9870	0.9032	0.9028	0.6826	0.8393	Layer fc1, fc2, fc_g1, fc_g2, fc1_xt (fully connected layer after concatenation, and on smiles block and protein block)

Abbreviation: MSE, mean squared error.

*Note*: The results of the experiment within the third scenario (except for train loss value) outperformed the experiments with the other scenarios. Each scenario details are described in the description column.

### 2.4 GraphDTA layer experiments

Based on Table 8, the first change added two FC layers immediately before the output layer. This was to enrich the feature representation before predicting the final output. The train loss increases more than GCN significantly to 0.2663, while the validation loss is not much lower (0.8417 vs. 0.8450) after hyperparameter tuning. Furthermore, the MSE was also marginally higher (0.8094 vs. 0.8059), which suggests that this added FC layer does not bring substantial benefits to the prediction accuracy. Finally, two experiments were designed to enhance the model’s ability to capture information from the molecular graph and the protein sequence. The experiments added one GCNConv layer, one Conv1D layer, and one FC layer. In the output, we can see that the model underwent overfitting, as the validation loss increased heavily to 1.0233, and the MSE worsened to 0.9074. Even though the train loss is low (0.1845), the large gap between train and validation loss clearly shows that the model is too complex and cannot generalize very well.

**TABLE 8:** GraphDTA layer experiments.

Epoch	Train loss	Val loss	Best MSE	Best *R* ^2^	Best CI	Description
1000	0.2663	**0.8417**	**0.8094**	**0.7155**	**0.8424**	Add 2 fc layers before the output layer
1000	0.1845	1.0233	0.9074	0.6810	0.8317	Add 1 GCNConv, Conv1d, and fc3 Linear layer
1000	**0.1667**	0.9402	0.8820	0.6899	0.8372	Reduce the number of GCNConv layers from 3 to 2, removing 1 layer in the FC layer

Abbreviation: MSE, mean squared error.

*Note*: The results of the experiment within the first scenario (except for train loss value) outperformed the experiments with the other scenarios. Each scenario details are described in the description column. The best MSE, best R2, and best CI values in the first experiment should be bold as well, considering these values also outperformed the other experiments values.

As demonstrated by the experimental results, GCN after hyperparameter tuning is still the best model according to MSE, *R* ^2^, and CI values. By adding new layers (both FC and GCNConv), the model becomes increasingly complex, which leads the model to overlearn, which does not necessarily improve the accuracy of the model significantly. Less overfitting is achieved by simplifying the model after reducing the number of layers. However, its performance would not be optimal compared to the perfectly tuned GCN. Improving model performance is best handled with additional regularization (e.g., higher dropout or batch normalization) rather than increasing or decreasing the number of layers in the overall architecture.

### 2.5 BiGraph‐DTA

**FIGURE 1:**
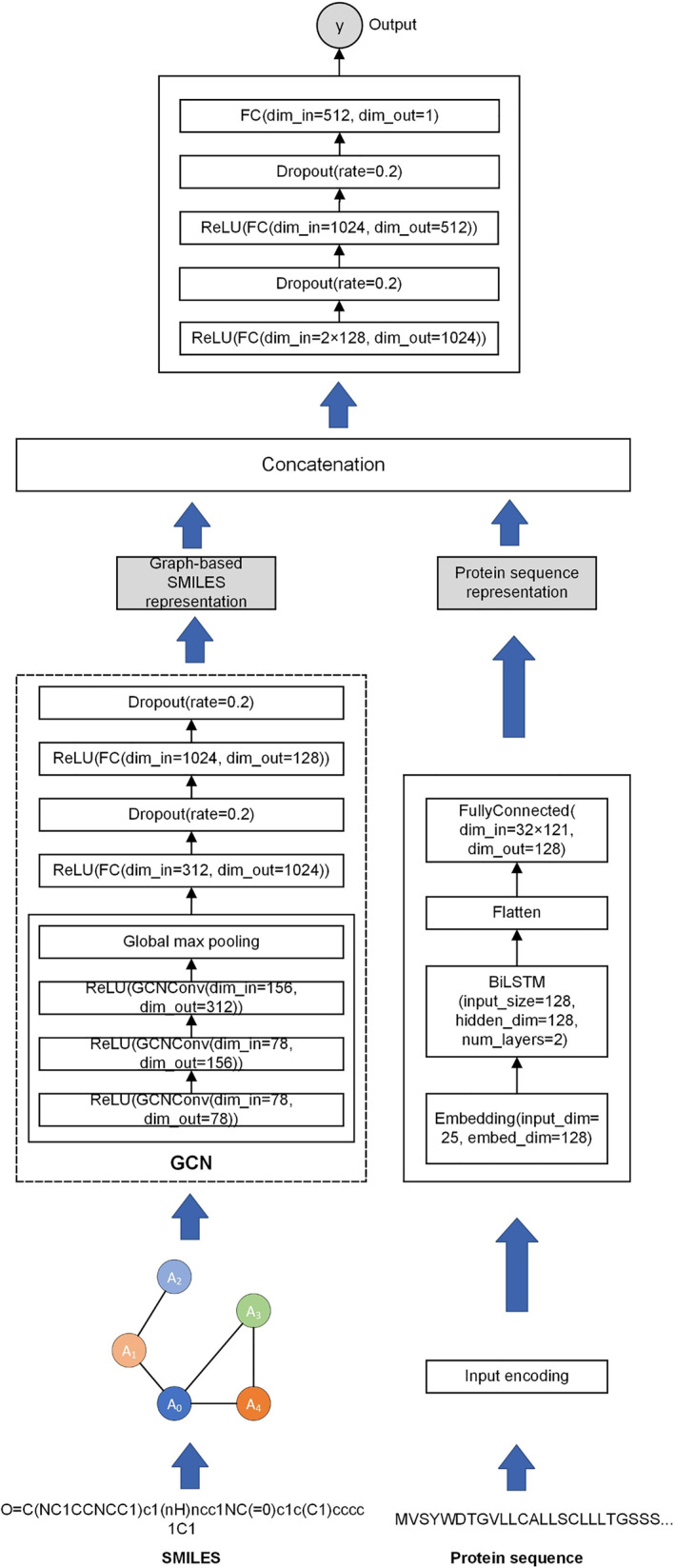
BiGraph‐DTA architecture. Graph convolutional network (GCN) and bidirectional long short‐term memory (BiLSTM) are applied for processing two‐dimensional (2D) graphs for drug molecules and text‐based (1D) protein sequences, respectively. Each representation is concatenated and passed into a fully connected neural network and dense layers to predict the final affinity score.

In Figure 1, the architecture of the DL‐based DTA prediction model, BiGraph‐DTA, is applied to predict molecule structure representation (SMILE code) and protein sequence: Each of them is sent through several layers of GCN with ReLU activations and then to global max pooling to get features. Features are tuned more and avoid overfitting using FC layers with dropout regularization.

Unlike CNN, we take an embedding layer feature vector for the protein sequence put into the BiLSTM network and learn to synthesize dependencies and contextual information. BiLSTM outputs are protein feature(s) that are flattened and then passed through a FC layer.

After obtaining the feature representations from both the SMILES and protein sequences, the model concatenates these features and processes them through a FC neural network to predict the final affinity score. The final network consists of multiple dense layers with ReLU activations, dropout layers, and a final FC layer that outputs a single prediction value. The BiGraph‐DTA model effectively captures structural and sequential dependencies from both molecular graphs and protein sequences, enabling improved drug–target interaction predictions.

**FIGURE 2:**
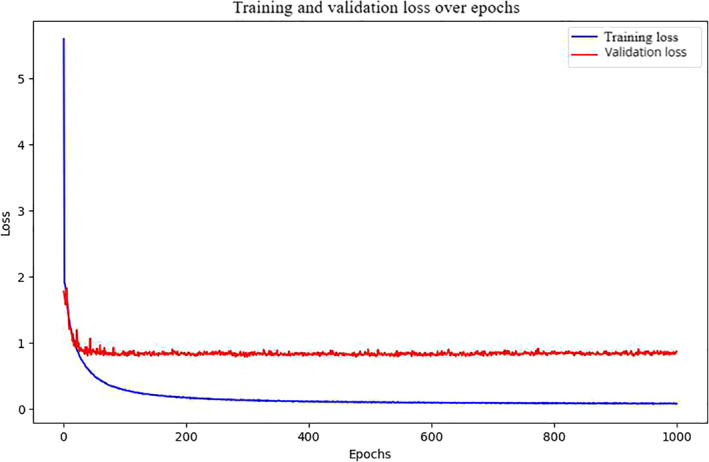
Training and validation loss on BiGraph‐DTA with hepatoprotector dataset. Although the model is learning well during the initial period, the continuation of training loss decrease shows that the model is not generalizing well.

Figure 2 is the training and validation loss from the BiGraph‐DTA hepatoprotector model for 1000 epochs (a substantial decrease in both loss functions over the first epoch means it is learning well during this period). The training loss continues to decrease while the validation loss plateaus at a greater value, suggesting that the model is not generalizing [34, 35].

**FIGURE 3:**
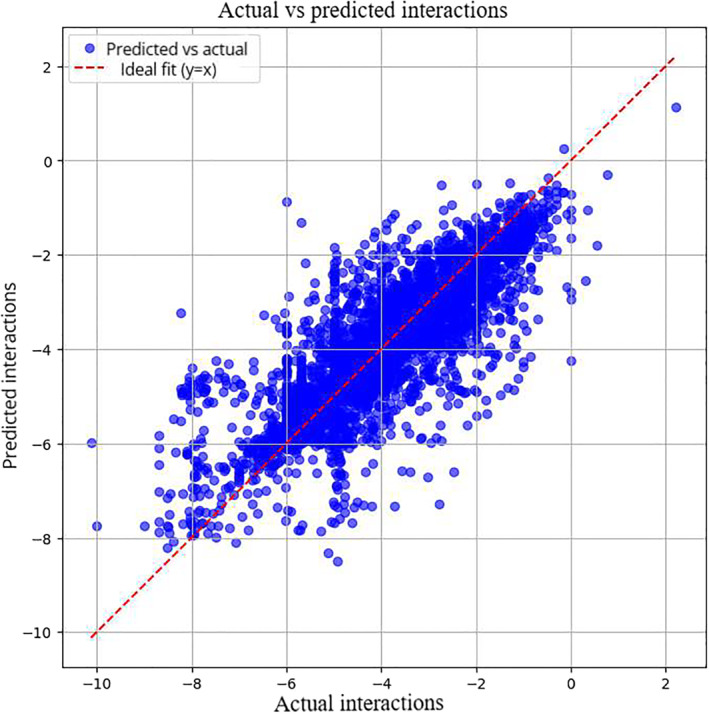
Prediction scatter plot on hepatoprotector dataset. The dots are not uniformly scattered in each small section of the diagonal. The model knows the rough direction but does not predict what the relative binding affinity is in any specific area.

The scatter plot displays the relationship and overall upward trend between expected and actual values in Figure 3. Even though the middle region is a very crowded area for our data, the dots are not uniformly scattered in each small section of the diagonal. That means the model knows the rough direction but does not predict what the relative binding affinity is in any specific area. This clustering can show a need for further tuning of a model or feature, insufficient training data for certain ranges of values, or overfitting.

Following that, Figure 3 shows more validation to support the suitability of BiLSTM for protein sequences, this time a comparison between traditional GCN and GCN combining BiLSTM (instead of 1D‐CNN as used by GraphDTA) is performed. In having to propose this change, the goal is that BiLSTM will be able to better capture individual features of protein sequences in a manner that allows for improved accuracy in the prediction of such drug–protein interactions. The standard GCN model demonstrated satisfactory performance. The prediction error rate MSE was 0.8059, and a relatively high correlation was observed between the predicted and original values *R* ^2^ of 0.7167. The model demonstrated a commendable capacity to predict the order of interaction strength, exhibiting a CI of 0.8423. Based on the results, we basically conclude that the model predicts binding affinity between protein and drug fairly and has a reasonable error range with relatively high correlation between predicted value versus true value.

Table 9 indicates that adding a BiLSTM network to extract features from protein sequences with a hidden dimension of 128 significantly enhanced the model performance metrics. This improvement is shown by the lower train loss (0.0808), validation loss (0.8712), and maximum likelihood estimation MSE (0.7885). In addition, the model showed that the root mean square error (*R* ^2^) was 0.7208 and that the CI was 0.8508, indicating enhanced accuracy of the model in the prediction. This indicates that based on these results, the model with BiLSTM makes more accurate predictions than the conventional GCN. The decreasing value of the MSE and the increasing values of *R* ^2^ and the coefficient of determination (*R* ^2^) indicate that this model is better at modeling the relationship between drug and protein features. However, the higher train loss and validation loss compared to the standard GCN indicate that the model may struggle to stabilize or require regularization to prevent overfitting.

**TABLE 9:** New architecture with BiLSTM evaluation table.

Epoch	Train loss	Val loss	Best MSE	Best *R* ^2^	Best CI	Description
1000	**0.0808**	**0.8712**	**0.7885**	**0.7208**	**0.8508**	BiLSTM dim 128
1000	1.8766	1.8866	1.8818	0.3384	0.6917	BiLSTM dim 128, cross attention + concatenation
1000	0.0825	0.9171	0.8132	0.7141	0.8429	BiLSTM dim 128, co‐ attention + concatenation

Abbreviation: MSE, mean squared error.

*Note*: All results of the experiment within the first scenario outperformed the experiment with the other scenarios. Each scenario details are described in the description column. The train loss and val loss in the first scenario experiment should be bold as well, considering these values also outperformed the other experiment values.

The architecture of the Tang’s model, 1D‐CNN, was first designed for protein sequence feature extraction, which has been shown to be quite effective in capturing local spatial patterns, as well as feature hierarchies in a sequential manner. However, CNNs struggle to capture long‐term dependencies within protein sequences. The difference is that BiLSTM employs a memory mechanism that can capture long‐term information. This is advantageous in the case of protein sequences since the model considers direct representation of information from two directions, in contrast to using only left context or right context. Overall, the GraphDTA supplemented with BiLSTM was established as the best candidate for replacing the GCN in GraphDTA because of its effectiveness in improving the accuracy and stability of solutions (based on metrics of MSE, *R* ^2^, and CI). But the increase in train loss and validation loss indicates that the model possibly requires more tuning in terms of hyperparameter tuning and regularization for steadier convergence.

Table 10 shows that by augmenting the BiLSTM architecture with cross attention and co‐attention mechanisms to further improve feature extraction from protein sequences. Adding cross attention in the BiLSTM architecture was able to deliver a training loss at 1.8766, a validation loss at 1.8866, an MSE at 1.8818, an *R* ^2^ at 0.3384, and a CI at 0.6917. These results show a significant drop in performance compared to the BiLSTM ablation with attention, which may be due to the model struggling to accommodate the added complexity from cross attention.

**TABLE 10:** New architecture with GraphDTA graph convolutional network evaluation table.

Epoch	Train loss	Val loss	Best MSE	Best *R* ^2^	Best CI	Description
1000	**0.0952**	**0.9053**	**0.7955**	**0.7203**	**0.8443**	Cross attention + concatenation
1000	0.1103	0.9305	0.8676	0.6950	0.8397	Co‐attention + concatenation

Abbreviation: MSE, mean squared error.

*Note*: All results of the experiment within the first scenario outperformed the experiment with the other scenarios. Each scenario details are described in the description column. The train loss and val loss in the first scenario experiment should be bold as well, considering these values also outperformed the other experiment values.

The results for BiLSTM + co‐attention model showed a training loss of 0.0825, a validation loss of 0.9171, an MSE of 0.8132, and for an *R* ^2^ of 0.7141 and a CI of 0.8429. It slightly reduces performance when compared to the BiLSTM without attention but stays competitive with the standard GCN. The implication of the results is that the continued application of cross attention does not help improve the model but rather results in a significant drop in the model performance. The architecture with co‐attention is relatively stable, although it has worse performance than the BiLSTM without attention.

From Table 11, an extensive analysis of experimental results highlights that BiGraph‐DTA with BiLSTM provides superior performance over alternative approaches, as evidenced in Table 7. When comparing the metrics of the standard GCN with the above model, the MSE, *R* ^2^, and CI are given by MSE of the model is 0.7885, *R* ^2^ at 0.7208, and CI at 0.8508 against MSE in GCN is 0.8059, *R* ^2^ of 0.7167, and CI of 0.8423. Such observations confirm the assumption that BiLSTM is more effective than the 1D‐CNN model during the extraction of features from the protein sequences because it can identify long‐time dependencies presented in protein sequences.

**TABLE 11:** Summary metrics evaluation from every scenario.

Method	Protein rep.	Compound rep.	MSE	*R* ^2^	CI
Hepatoprotector dataset					
GraphDTA (**GCN)**	1D	Graph	0.8059	0.7167	0.8423
GraphDTA (**GCN + SVR)**	1D	Graph	1.9373	0.3189	0.7016
GraphDTA (**GCN + RF regressor)**	1D	Graph	1.0725	0.6230	0.8083
GraphDTA (**GCN + XGBoost)**	1D	Graph	1.1224	0.6054	0.7990
DeepDTA	1D	1D	1.1355	0.6008	0.6008
Random forest	1D	1D	1.3490	0.3210	0.7200
XGBoost	1D	1D	1.1270	0.4250	0.7610
GraphDTA (transfer learning with KIBA dataset)	1D	Graph	0.7964	0.7200	0.8417
GraphDTA (transfer learning with Davis dataset)	1D	Graph	0.8129	0.7142	0.8466
GraphDTA (regularization with Lasso)	1D	Graph	0.8946	0.6855	0.8384
GraphDTA (regularization with Ridge)	1D	Graph	0.9079	0.6808	0.8424
GraphDTA (regularization with ElasticNET)	1D	Graph	0.8888	0.6875	0.8431
GraphDTA (experiment layer)	1D	Graph	0.8094	0.7155	0.8424
BiGraph‐DTA	1D	Graph	**0.7885**	**0.7208**	**0.8508**
BiGraph‐DTA (cross attention + concatenation)	1D	Graph	0.7955	0.7203	0.8443

Abbreviations: DTA, drug–target affinity; GCN, graph convolutional network; MSE, mean squared error.

*Note*: The results of the experiment using BiGraph‐DTA method outperformed the experiments with the other method.

In comparison to DL methods, ML approaches (including support vector regression, random forest, and extreme gradient boosting) that rely on GCN features still performed poorly. Results showed that, of the methods, GCN + RF regressor performed best, having an MSE of 1.0725 and an *R* ^2^ of 0.6230. However, it was still far behind DL models.

Technical details regularization methods (Lasso, Ridge, and ElasticNet) slightly improved performance (but only slightly) while maintaining some stability of the model. At the same time, transfer learning leveraging the KIBA and Davis datasets proved useful as well, with KIBA showing marginally better performance than the basic GCN model (MSE = 0.7964, *R* ^2^ = 0.7200, CI = 0.8417). This result suggests that transfer learning might be a useful strategy in some settings.

To validate the effectiveness of BiGraph‐DTA, we compared its performance with several state‐of‐the‐art models, including GraphDTA, DeepDTA, random forest, and XGBoost. The following Table 12 summarizes the results of this comparison:

**TABLE 12:** Models comparison.

Model	MSE	*R* ^2^	CI	Strength	Limitation
BiGraph‐DTA	**0.7885**	**0.7208**	**0.8508**	Combines GCN and BiLSTM to capture long range protein sequence dependencies	Requires more training data for stabilization, long training time
GraphDTA (GCN)	0.8059	0.7167	0.8423	Fast and effective on large datasets	Struggles with domain‐specific datasets
DeepDTA (CNN)	1.1355	0.6008	0.6008	Simple CNN‐based architecture	Fails to capture long‐range protein dependencies
Random forest	1.3490	0.3210	0.7200	Fast and interpretable	Poor performance on complex DTA prediction
XGBoost	1.1270	0.4250	0.7610	Efficient for regression tasks	Less effective on graph‐structured data

Abbreviations: CNN, convolutional neural network; DTA, drug–target affinity; GCN, graph convolutional network; MSE, mean squared error.

*Note*: The results of the experiment using BiGraph‐DTA outperformed the experiments with the other models.

From Table 12, it is evident that BiGraph‐DTA demonstrates the best performance due to the values of MSE, *R* ^2^, and CI. The superior performance of BiGraph‐DTA can be attributed to its unique ability to integrate GCN for drug structure representation and BiLSTM for protein sequence processing. This integration allows BiGraph‐DTA to capture both the structural and sequential components of drug–target interactions, which traditional models fail to do.

Overall, BiGraph‐DTA can outdo standard GCN, GraphDTA, transfer learning, and regularization, as well as other GCN‐based ML methods. Results indicate the advantage of using BiLSTM rather than 1D‐CNN for protein sequence feature extraction and show improved result for drug–proteins interaction prediction model performance.

## 3 DISCUSSION

Our model has proven effective in predicting hepatoprotective ligand–protein interactions. Meanwhile, accurate identification of potential therapeutic targets for liver disease can have profound implications for the pharmaceutical and health industries. This way, the drug discovery process may be considerably optimized, allowing them to pinpoint new treatment candidates much sooner and cheaper. These results will improve treatment outcomes for patients and reduce the burden of liver disease on individuals and society.

Because of the absence of reliable evidence regarding liver toxicity, data were collected and selected at a rapid pace. The dataset was obtained from the ChEMBL database: a large publicly accessible database of bioassay results for large numbers of compounds that have been screened against various target proteins and can include the IC50 type. The target proteins were selected considering their relation to antifibrotic, anti‐inflammatory, antioxidant, anti‐apoptotic, and various other properties to evaluate their capability as hepatoprotectors. One of the main limitations of the study is only therapeutical candidates for liver disease; no mechanism of action or prediction of the adverse reactions is presented. Clinical studies are ultimately required to assess the efficacy and safety of therapeutic candidates by further deciphering the pathways and targets implicated in liver disease. Additionally, the precision medicine approaches that could be pursued for liver disease therapy considering putative genetic and environmental determinants of treatment response should be explored. By doing so, we can overcome these limitations, identify novel targets for the next stage of discovery, and help advance the drug development process to treat liver disease and potentially enhance care outcomes for patients. This may result in interventions that are more focused and efficient. That could have facilitated the drug search by identifying the best therapeutic option and helping to avoid squandering money and years. Additionally, the applicability of our method across independent datasets indicates that our findings may generalize not just about liver disease but also about other diseases. It would transform drug discovery and testing, and be a huge leap for many diseases.

Interestingly, this observation not only signifies that the utilization of data dimensionality is a principal feature for training the DL models but also suggests that prediction of drug–target interaction needs a further level of both representation and abstraction in data to infer complex addictiveness for the protein as well as the molecules. Therefore, it might be possible to bridge the performance gap by either enlarging the hepatoprotector dataset with more compounds or using more sophisticated data augmentation or transfer learning methods.

Our proposed model, BiGraph‐DTA, extends the GraphDTA architecture to enhance the prediction performance of DTA tasks. The main improvement of our approach concerns the handling of protein sequences; we exchanged the original 1D CNN‐based feature extraction strategy for a Bi‐LSTM network. This enables the model to better capture long‐range dependencies and contextual relationships within protein sequences. Better sequential buried features are learned through the multi‐layered structure of the Bi‐LSTM model [36]. We experimented with several approaches and evaluation metrics and ultimately chose a GCN for molecular SMILES and Bi‐LSTM for FASTA protein data feature extraction. Based on experimental results, this architectural improvement achieves better evaluation metrics than the predictive power of the original GraphDTA architecture.

The results of experiments indicate that the GCN model outperforms other models such as DeepDTA, random forest, and XGBoost on the hepatoprotector dataset. In the hepatoprotector dataset, GCN yielded the lowest mean squared error (MSE = 0.919) and the highest (CI = 0.790), outperforming that observed using DeepDTA (MSE = 1.175, CI = 0.409), random forest (MSE = 1.349, CI = 0.720), and extreme gradient boosting (XGBoost) (MSE = 1.127, CI = 0.761). This means the models have a strong capacity to learn both how to effectively represent molecular graphs as well as sequence‐based properties of proteins.

However, the GCN performance on the hepatoprotector dataset is lower than the larger datasets (KIBA, Davis). To the best of our knowledge, GCN performed at a MSE of 0.139 with a CI of 0.889 on the KIBA dataset, while optimal performance for the Davis dataset was MSE = 0.254 and CI = 0.880 [15]. This shows how crucial the size of the dataset is for a good model.

Although the corresponding GCN model performed better than other models, such as DeepDTA, random forest, and XGBoost for hepatoprotector, it has been shown that the overall accuracy was much lower than GCN on KIBA and Davis (MSE = 0.919 vs. CI = 0.790). On the KIBA dataset, MSE is 0.139 and CI is 0.889 and on the Davis dataset, MSE is 0.254 and CI is 0.880; GCN outperformed all the competitors.

This difference is mainly because of the scale of the datasets, unlike the widely adopted KIBA and Davis datasets containing hundreds of thousands of samples [37, 38]. Deep models with higher data depth provide richer features, allowing for a higher level and more diverse representation of the input space, leading to better accuracy and generalization. The small count samples in the hepatoprotector dataset restrict diversity in the training data, impairing the model’s capability to generalize unseen instances, which naturally lead to poor performance measures.

Many research studies have shown that smaller sample sizes can lead to reduced prediction accuracy, which causes small sample sizes (i.e., number of classes < 20 (*n* < 20)) to give incorrect results [39]. Predictors that have larger datasets enable easier training of models [40]. It has therefore come to our attention to develop a GCN technology that has a meaningful potential for predicting hepatoprotective medicines as well as their interactions with the therapeutic targets. With this new approach, scientists could improve target identification for potential pharmaceutical candidates and prescribe treatment in a way that fits unique patient circumstances. Thus, this exploration of new methodologies certainly sounds imperative, as enhancing the personalization of the patient care outcome and challenging the usual ways would certainly be of benefit in our quest for effective treatments of liver disease. By conducting further research and collaborating, significant improvements in liver disease treatment, and consequently patient prognosis, can be achieved.

This was the best result of all the experiments, as the GCN model reports an MSE of 0.8059, an *R* ^2^ of 0.7167, and a CI of 0.8423 after hyperparameters are tuned (without regularization). The use of Lasso and Ridge regularization resulted in performance improvements in a few cases, but their tuning did not clearly outperform GCN. ElasticNet yielded more robust results but did not improve on the baseline, which had no regularization. In conclusion, GCN post hyperparameter tuning is rated as the best method. But, when regularization is included, ElasticNet on the fc1 and fc2 layers becomes the best candidate and produces competitive results without major change of performance.

Both transfer learning experiments with the KIBA and Davis datasets lead to the conclusion that once a proper search for hyperparameters is made, the GCN model always returns optimal results (MSE = 0.8059, *R* ^2^ = 0.7167, CI = 0.8423). The best transfer learning case scenario is as follows: the KIBA dataset used without freezing of layers: MSE = 0.7964, *R* ^2^ = 0.7200, and CI = 0.8417. This method produces results slightly better (after tuning) than the GCN model. Using the Davis dataset without freezing any layers (MSE = 0.8129, *R* ^2^ = 0.7142, CI = 0.8466), the resulting performance is like GCN’s, once tuned. Apart from that, if you freeze some of the layers or add new layers without the appropriate adaptation strategy, the model’s performance is also degraded, making MSE higher and *R* ^2^ lower. This discovery highlights the need for proper implementation to make sure that the transfer learning worked. Through the power of transfer learning, especially when avoiding layer freezing, the KIBA dataset outperformed GCN when hyperparameters were tuned.

To improve feature extraction from protein sequences further, cross attention and co‐attention mechanisms were added to the BiLSTM architecture. BiLSTM + cross attention resulted in a training loss of 1.8766 and a validation loss of 1.8866, with MSE = 1.8818, *R* ^2^ = 0.3384, and CI = 0.6917. These results show a significant decrease in performance compared to BiLSTM without attention, possibly due to the model struggling to handle the increased complexity of cross‐attention. BiLSTM + co‐attention resulted in a training loss of 0.0825 and validation loss of 0.9171, with MSE = 0.8132, *R* ^2^ = 0.7141, and CI = 0.8429. Although there is a slight decrease in performance compared to BiLSTM without attention, the metrics are still in a competitive range with the standard GCN. These results indicate that the addition of cross attention does not improve the performance of the model and, in fact, significantly decreases its accuracy. However, co‐attention still provides stable performance, although it does not outperform BiLSTM with no attention mechanism.

However, the BiGraph‐DTA model does not come without limitations, and there are cases where the model might perform poorly. One major issue is that the approach relies on high‐quality large datasets. BiGraph DTA is intended to have strong generalization capabilities even for domain‐specific datasets, yet it may not perform well for a dataset that is too small or not diverse. Unsurprisingly, if the training data cover or under‐sample the drug–target interaction poorly, the model does not adequately learn the complex interaction and makes incorrect predictions.

Another limitation is related to the well‐distributed molecular structures assumption. BiGraph‐DTA uses drug graphs and depends on the graph representation of drugs, assuming that the structural information is perfect and is not missing. If the input was noisy or incomplete (e.g., because of lacking experimental data about drug properties), the model might misunderstand the molecular structure and make erroneous predictions. It can be a big problem in early drug discovery when your molecular data might be limited or noisy.

The BiLSTM model is powerful for modeling sequential dependencies in protein sequences, but it is not immune to the longer‐range dependencies from larger protein structures or more complicated interactions. For protein sequences with large variations or mutations that are not captured during training, BiGraph‐DTA is likely to fail to make accurate predictions, which can result in either false positives or negatives. Additionally, BiLSTM requires significantly longer training times compared to CNNs, as it processes sequences iteratively and captures long‐range dependencies. This added complexity and extended training time can make it challenging to scale the model for large datasets or time‐sensitive applications.

Furthermore, BiGraph‐DTA might have limitations in multi‐target drugs that interacts with a wide range of proteins. Because the model is trained on one drug and one target protein only, its performance can deteriorate when extending the dataset to a multi‐target dataset or complicated drug mechanisms. This could restrict its usefulness for drug discovery in general, where polypharmacology (the ability of a drug to affect multiple targets) is common.

Although BiGraph‐DTA has achieved considerable success in DTA’s prediction, there is still space for improvement. In the future we will further improve the accuracy of our model by the combination of geometry optimization (GO) along with active learning strategies, which may also refine to some extent both the drug and protein representations. GO is focusing on finding the optimized 3D coordinates of atoms (or other particles) in a molecule to become the local minimum of energy, generally using either quantum mechanical calculations or force fields. GO has been used in drug discovery to inspect molecular docking results and optimize molecular structures by minimizing their energy values for improved predictions of drug–target protein interactions [41, 42]. Through GO, better 3D structures are derived for the drug and target proteins, which makes the model more capable of learning the relative spatial positions and interaction potentials between the drug and proteins. This feature might be beneficial to the model’s predictions enhancement in systems where conformational states are essential to drug–target binding. This can be associated with molecular modeling techniques such as Ref. [31] to enhance the GNNs with 3D structural information of the protein–ligand binding poses for better prediction of drug–target interactions.

On the other hand, active learning is a ML technique that annotates the most informative items in an unlabeled dataset throughout the course of training [43, 44]. In the DTA prediction area, we can apply active learning to continuously select drug–target pairs that are uncertain and difficult to predict by the model, so the model is able to learn from the most difficult samples. This might help the model learn better by concentrating its learning effort on the places where it can be most improved. Through incorporating active learning, we can iteratively boost the model’s performance by minimizing uncertainty and increasing the effectiveness of the training. Methods from the active learning scheme as utilized by Nguyen [15], which can further be employed for DTA prediction for efficient search space refinement during model training to select more relevant interactions automatically, as well as refine the effectiveness of the learning process.

By incorporating our proposed framework BiGraph‐DTA with GO and active learning, we expect it could obtain better precision prediction for interactions and have better generalization to variety drug–target pairs, especially in the complex datasets and multi‐drugs and target scenarios.

## 4 CONCLUSION

BiGraph‐DTA is a new hybrid DL method created for the DTA prediction (e.g., hepatoprotective agents). Incorporating both graph structure and sequence features with bidirectional LSTM, this method can mine both the structural and sequential information in drug–target interactions, leading to significant improvement in both predictive performance and generalization capabilities against the prior models. Unlike previous works, which mainly use general datasets such as Davis and KIBA, we employ a hepatoprotective‐specific dataset to test BiGraph‐DTA in this domain. Although previous methods such as GraphDTA, DeepDTA, and classical ML models have made great strides in the prediction of DTA, they may lack extensibility when faced with domain‐driven data such as hepatoprotective drug data. BiGraph‐DTA is designed to tackle these issues by fusing the graph‐level learning of GCN and the depth of sequence modeling in the BiLSTM, which provides a robust and accurate approach to predict drug–target interactions in liver protection.

Compared with the conventional models such as GCN, BiGraphDTA achieves substantially better performance on predicting target–ligand affinities, as indicated by the higher (MSE) of 0.7885, *R* ^2^ of 0.7208, and CI of 0.8508 than that of GCN (MSE = 0.8059, *R* ^2^ = 0.7167, CI = 0.8423). These conclusions can confirm our hypothesis that BiLSTM is superior to 1D‐CNN in protein sequence feature extraction due to capturing the long–long dependencies within protein sequences, and accordingly, the overall prediction performance is enhanced.

BiGraph‐DTA demonstrates significant potential for accelerating the drug development process, not only for hepatoprotective drugs but also for broader clinical applications. The model’s ability to integrate multi‐dimensional data representations, such as the two‐dimensional graph data for drug compounds (converted from SMILES code) and one‐dimensional data for target proteins, is a promising strategy for enhancing feature extraction. Incorporating additional features could further improve performance, and employing advanced computational techniques such as GCN could significantly streamline the drug discovery process. This could lead to the rapid development of superior therapies, reducing the time and cost traditionally associated with drug discovery, and leveraging advances in personalized medicine for a wide range of diseases.

Although we have obtained such inspiring results, our model has several limitations, especially on data quality, small‐sized datasets, and multi‐target interactions. Additional improvements to the model should be carried out to be able to apply it to a wider set of in silico drug discovery applications. Recognition of these limitations is essential for model refinement and increasing its clinical applicability.

Moving forward, GO and active learning will be incorporated as a part of our future work to improve the accuracy of molecular structure to increase efficiency in learning the optimal binding between drug and protein. Although these methods improve predictability, especially for multi‐target drug discovery and the accurate prediction of complex drug–protein interactions, using more sophisticated drug compound and target protein representations can be done. Such advancements will not only increase the utility of BiGraph‐DTA in drug discovery pipelines but also support the development of more effective hepatoprotective agents and therapies for other diseases.

## 5 MATERIALS AND METHODS

### 5.1 Dataset

The suggested model was assessed using a hepatoprotective dataset collected from the CheMBL. ChEMBL is a manually curated database of bioactive molecules with drug‐like properties. It brings together chemical, bioactivity, and genomic data to aid the translation of genomic information into effective new drugs [45]. The hepatoprotective agents mostly correlate with compounds demonstrated to exhibit antifibrotic, anti‐inflammatory, antisteatotic, anti‐apoptotic, cell survival, and antiviral activities by interacting with various molecular targets and signaling pathways [46]. Therefore, several target proteins related to these activities with sufficient data availability were selected. The dataset concentrated on drug compounds interacting with the hepatoprotective target protein, including cyclooxygenase‐2 (ChEMBL230), carnitine O‐palmitoyltransferase 1, liver isoform (CHEMBL1293194), cholesterol acyltransferase (ChEMBL6141), glutaminase liver isoform, mitochondrial (CHEMBL4105730), liver glycogen phosphorylase (ChEMBL2568), protein kinase C alpha (ChEMBL299), matrix metalloproteinase‐2 (ChEMBL333), TNF‐alpha (ChEMBL1825), pyruvate oxidase (ChEMBL3380), cytochrome P450 2E1 (ChEMBL5281), MAP kinase p38 (ChEMBL2094115), heat shock protein HSP90 (ChEMBL2095165), HMG‐CoA reductase (ChEMBL402), fatty acid synthase (ChEMBL4158), Acyl‐CoA desaturase (ChEMBL5555), TGF‐beta receptor type I (ChEMBL4439), and hypoxia‐inducible factor 1 alpha (ChEMBL4261).

In our proposed model, protein sequences are represented as character sequences and padded to a fixed length of 1000. If the length is less than 1000, it is padded with zeros—the same logic applied to keeping the input dimensions intact. For sequences greater than 4,000, there will be truncation. SMILES representations form molecular structures treated similar to graphs, allowing the model to be agnostic to the length of the sequence and mitigating potential bias. Moreover, the representation of the molecules in a graph structure naturally contains information about molecular relationships, and parsers of varying lengths, which could be problematic in the string format, would not affect the performance of the model. After performing data preprocessing, a total of 21,421 interaction data points were obtained, containing the SMILES string representation, protein sequences, and corresponding IC50 values. The IC50 values were a conventional standard for the efficacy of a compound (half‐maximal inhibitory concentration).

**FIGURE 4:**
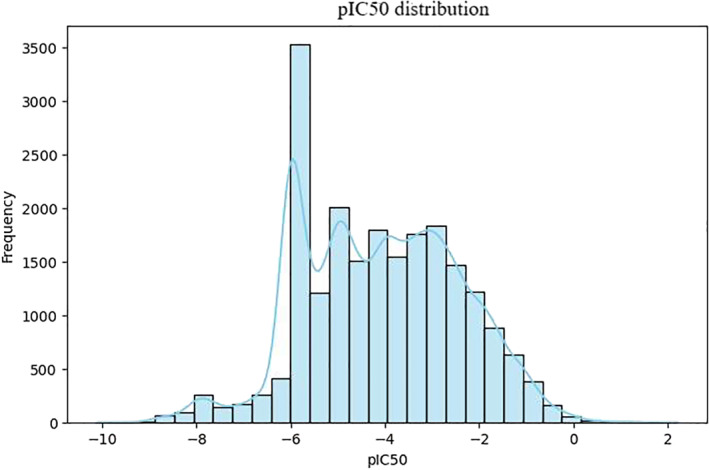
Distribution of pIC50 values (total interaction data points: 21,241 rows).

Figure 4 shows the distribution of pIC50 values. To minimize the enormous range of values the IC50 values from the dataset were transformed into log space (pIC50) using Equation (1). This makes data controllable by limiting extreme values while also rendering the data more interpretable. This approach is used in similar datasets to better represent the potency of compounds, as demonstrated in previous research [47]. The distribution is almost approximately normal, so regression modeling and statistical options can be applied to this data.

(1) $$p{\text{IC}}_{50}=-{\text{Iog}}_{10}\left({\text{IC}}_{50}\right).$$

To categorize the bioactivity of the compounds based on pIC50 value, the clustering stage was used. pIC50 is the negative logarithm of the IC50 value. The partitioning stage allows us to split a dataset into a few subsets for the purpose of training, validation, and testing. The latter aspect is crucial for assessing the performance of ML models and determining their ability to generalize. In this research an 80:20 ratio was used in which 80% was allocated for training and 20% was left over for external validation or testing. The model performance was assessed by means of 10‐fold cross‐validation on the training data (internal validation). The clustering and partitioning phases are most commonly used in feature and model selection in ML [48].

**FIGURE 5:**
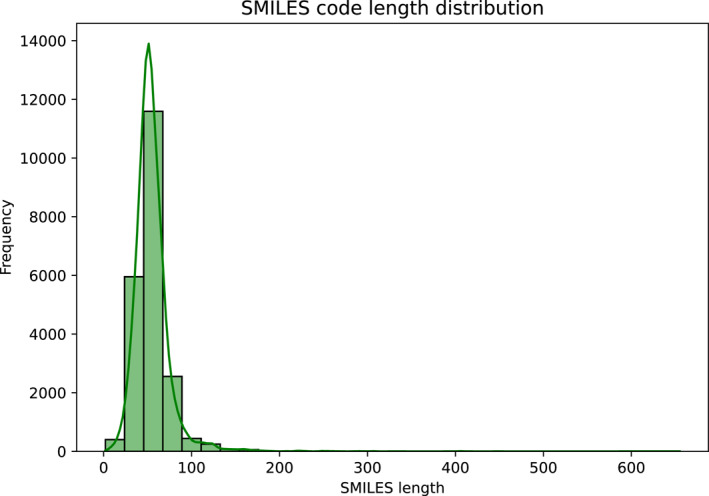
Distribution of SMILES code length.

From Figure 5, we obtained the SMILES strings for the compounds from PubChem; SMILES strings compactly represent structural information for chemical entities. The SMILES distributions code strings are represented in Figure 5, with a minimum length of 2 characters, a maximum length of 655 characters, and an average length of 55.20 characters.

**FIGURE 6:**
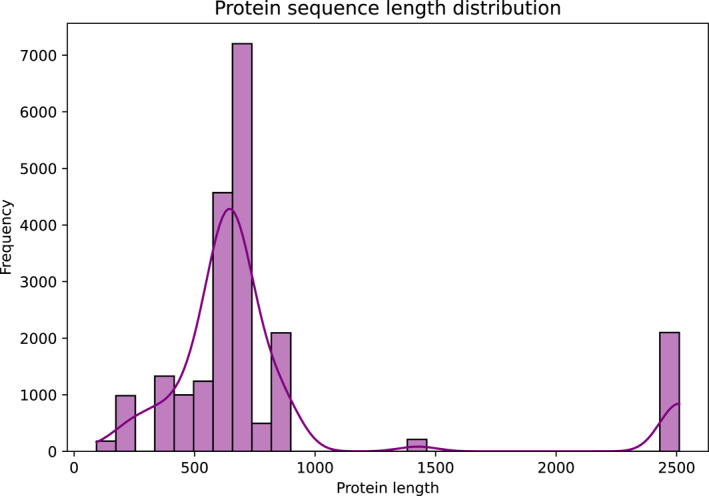
Distribution protein sequence length.

In Figure 6, protein sequences were obtained from the PubChem database using the target protein COX‐2 as an identifier. The distribution of protein sequence lengths in the dataset is shown in Figure 6. The lengths of the sequences have a minimum of 93 characters, a maximum of 2511 characters, and an average of 808,15 characters. These differences are likely to arise from the structural diversity of the compounds interacting with proteins, which enables a wide range for model learning. The dataset consists of sequences from 18 different target proteins, the most abundant of which is cyclooxygenase‐2 (COX‐2). This protein sequence with 4531 rows is the commonest protein in the data.

### 5.2 GCN‐based graph representation learning

**FIGURE 7:**
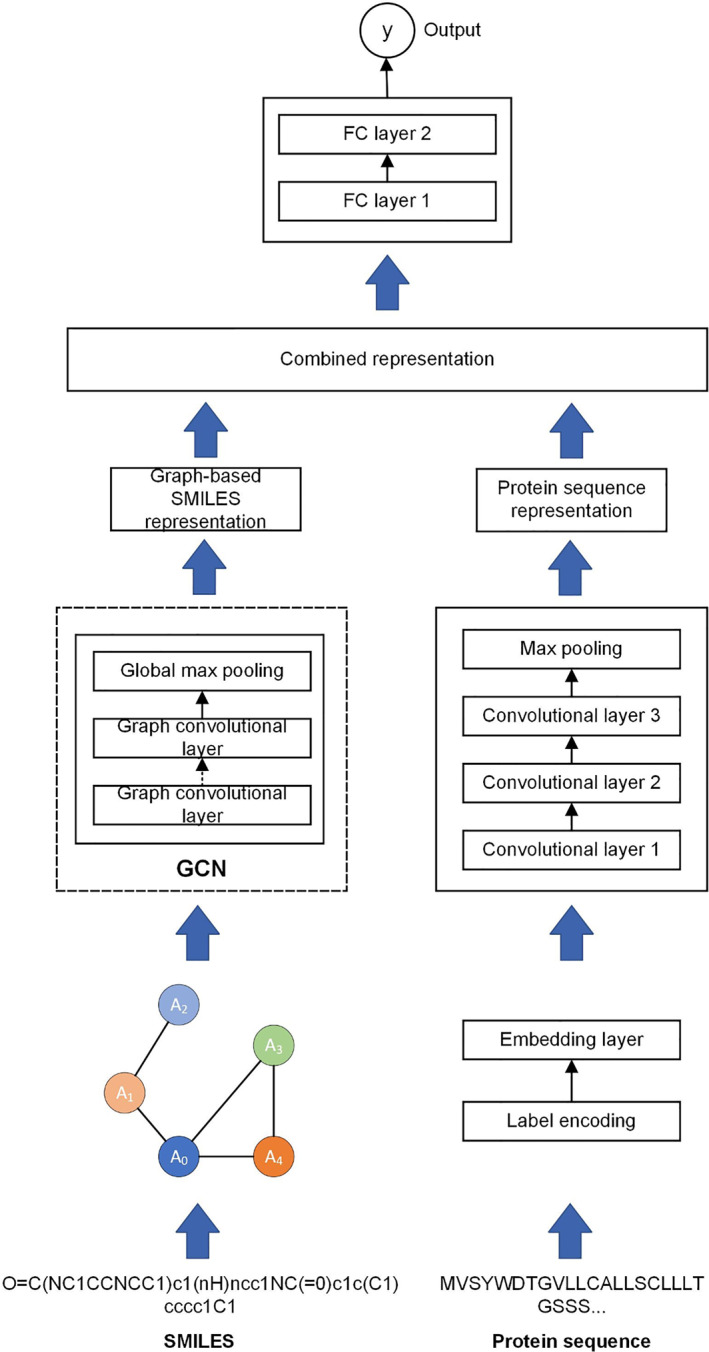
GraphDTA architecture [15]. Graph convolutional network (GCN) and convolutional neural network (CNN) are applied for processing two‐dimensional (2D) graphs for drug molecules and text‐based (1D) protein sequences, respectively. Each representation is concatenated and passed into a fully connected neural network and dense layers to predict the final affinity score.

From Figure 7, this study examines the prediction of the interaction level between drug and protein sequences, represented as a continuous value based on Figure 4. Each drug corresponds to a graph, and each protein is treated as an input sequence. To this end, drug representations are generated using the GCN model [49] based on drug graphs. The original GCN is built based on a semi‐supervised node classification problem, and the model learns node‐level feature vectors. In that context, a graph‐level representation is crucial for estimating drug–protein interactions for every drug. Common methods such as sum, average, and max pooling are utilized to derive the composite graph feature from the acquired node data, as shown in Equation (2),

(2) $$X\in R^{N\times C},$$

where *N* = |*V| A* ∈ $R^{N\times N}$, ∈ $R^{N\times F}$ [49], ensuring stability.

Consider a drug’s graph represented with *G* = (*V*; *E*), where *V* is a set of *N* nodes visualized by a C‐dimensional vector and *E* is a set of edges visualized through an adjacency matrix *A*, and take as input a multi‐layer GCN with a node feature input *X* ∈ $R^{N\times C\;}$(where *N* = |*V*|, and *C* is the number of features per node) and adjacency matrix *A* ∈ *R* ^*N×N*^, creating a node‐level output *Z* ∈ R ^*N×F*^ (where *F* is the number of output features per node). Whereas stability is guaranteed in this normalized form by Kipf and Welling [50], we can write the propagation rule in normalized form (the *F* coefficient indicates the number of features per node in the output), as shown in Equation (3),

(3) $$H^{\left(l+1\right)}=\sigma \left({\tilde{D}}^{-\frac{1}{2}}\;\tilde{A}{\tilde{D}}^{-\frac{1}{2}}H^{\left(l\right)}W^{\left(l\right)}\right),$$

where *Ã = A +* $I_{N}$ is the self‐connection added adjacency matrix of the undirected graph, *D̃*_*ii*_ *=* Σ_*j*_ *Ã*_*ij*_; $H^{\left(l\right)}\in R^{N\times D}$ is the activation matrix in the *l* ^th^ layer, $H^{\left(0\right)}$ = *X*, ***σ*** is an activation function, and *W* are learnable parameters, as shown in Equation (4),

(4) $$Z={\tilde{D}}^{-\frac{1}{2}}\;\tilde{A}{\tilde{D}}^{-\frac{1}{2}}\;X\Theta ,$$

where Θ ∈ RC × *F* is now a matrix of filter parameters and *Z* ∈ ℝ^*N*×*F*^ is the convolved signal matrix. This filtering operation has complexity O(|E|FC), as *Ã*, *X* can be efficiently implemented as a product of a sparse matrix with a dense matrix [49]. To derive the graph representation vector, a global max pooling layer is applied immediately after the final GCN layer. The GCN‐based model consists of three successive GCN layers, each utilizing a ReLU activation function; the global max pooling layer then aggregates the features to produce the final graph representation vector [15].

The pseudocode in Algorithm 1 summarizes the process of using a GCN‐based model for drug–protein interaction prediction, incorporating graph‐level representations from molecular graphs and protein features FC layers.

ALGORITHM 1 **Pseudocode of GCN model.**

1
 **Input**: *G* = (*V*, *E*, *X*) (molecular graph with nodes *V*, edges *E*, and features *X*),
 T (protein sequence), model parameters *θ*, number of epochs *e*
 **Output**: Trained model parameters *θ*
**1** Initialize model weights *θ* randomly.
**2** **for** epoch in 1,…, *e*:
**3**  **for** batch in training data:
**4**   Extract graph features *X*, *E*, *T* from batch
**5**   GCN Branch:
**6**    H_1_ ←ReLU(GCNConv(X,E))
**7**    H_2_ ←ReLU(GCNConv(H_1_,E))
**8**    H_3_ ←ReLU(GCNConv(H_2_,E))
**9**    H_g_ ←GMP(H_3_) (Global Max Pooling)
**10**   Protein Branch:
**11**    H_t_ ←Embed(T)
**12**    H_p_ ←Conv1D (H_t_)
**13**    Flatten H_p_
**14**   Combined Branch:
**15**    H_c_ ←concatenation(H_g_, H_p_)
**16**    Pass H_c_ through dense layers with ReLU and Dropout to compute y pred.
**17**   Compute loss
**18**   Update *θ* using gradient descent
**19**  **end for**
**20** **end for**
**21** Return trained parameters *θ*

### 5.3 DeepDTA

This study examines the prediction of the interaction level between drug and protein sequences, represented as a continuous value. Each medicine is coupled with a graph, and each protein is regarded as an input sequence. With this purpose in mind, the GCN model [49] is applied to learn drug representations on graphs of drugs. Nevertheless, the original GCN is predicated on a semi‐supervised node classification problem, wherein the model learns node‐level feature vectors. Graph‐level representation is set as necessary for each drug to assess drug–protein interactions. Popular approaches, including sum, average, and max pooling are applied to derive the aggregate graph feature from the learned node features [51].

**FIGURE 8:**
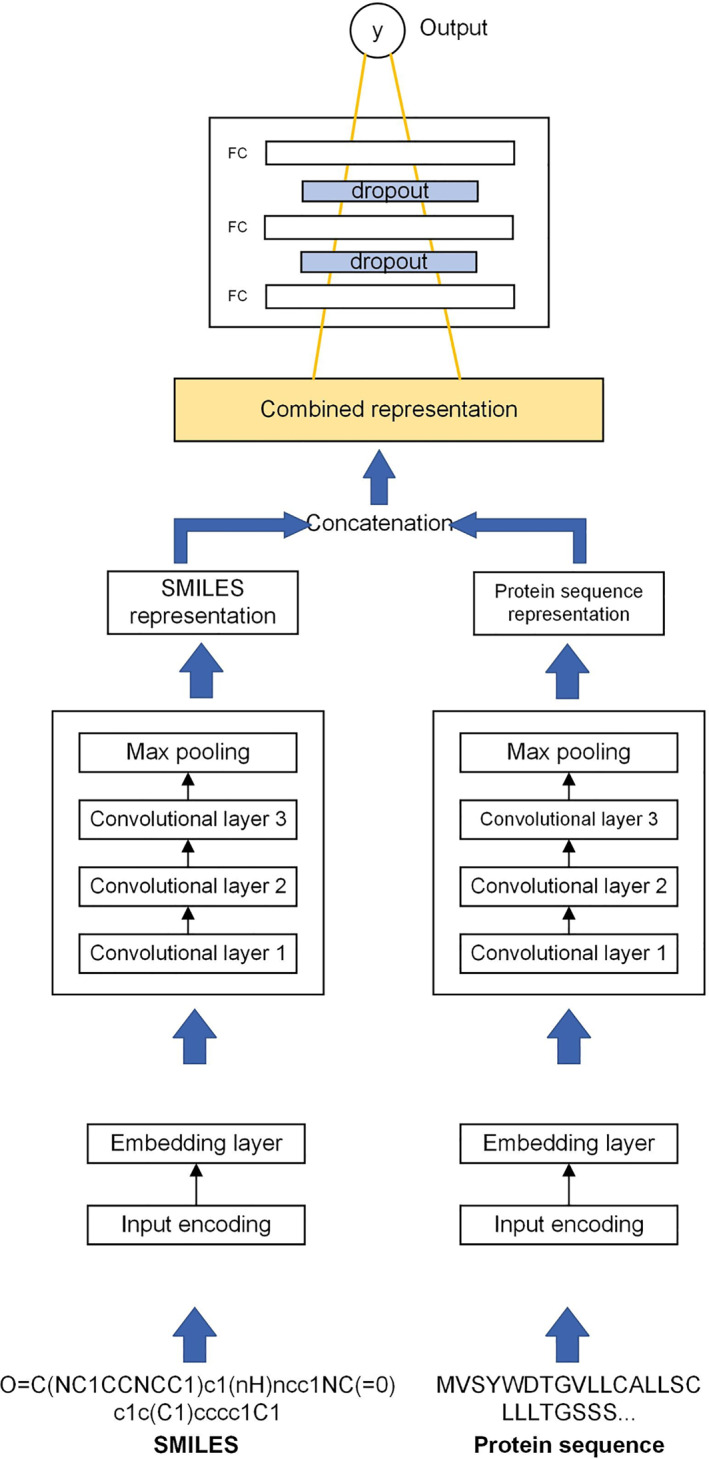
DeepDTA architecture [51]. Convolutional neural networks (CNN) are applied for text‐based (1D) drug molecules and text‐based (1D) protein sequences, respectively. Each representation is concatenated and passed into a fully connected neural network and dense layers to predict the final affinity score.

A CNN‐based prediction model was built as shown in Figure 8, consisting of two different CNN blocks for extracting representations from SMILES strings (C2‐6) and protein sequences (C2‐7). Each block was made up of three sequential 1D convolutional layers, with the second having twice as many filters as the first, and the third using three times more filters than the first layer. After the convolutional layers, a max‐pooling layer was applied.

The features from the max‐pooling layers were concatenation and then passed through three FC layers that together were termed deep drug–target binding affinity prediction (DeepDTA). The first two FC layers were composed of 1024 nodes each, followed by dropout layers with a 0.1 rate to prevent overfitting by deactivating some neurons during training [52]. A 512‐node FC layer was added after the previous one, followed by the output layer. Figure 8 gives a graphic representation of the proposed model with the included two CNN blocks.

The rectified linear unit (ReLU) was the activation function, specified as *g*(*x*) = Max (0, *x*), which is widely used in DL [53]. Log loss was used in training and optimization, where the goal was to minimize the distance between predicted and actual values. MSE was applied as a loss function because this is a regression job; here, *P* is the predicted values, *Y* is the ground truth outputs, and *n* is the number of samples in Equation (5).

(5) $$\text{MSE}=\frac{1}{n}\sum_{i=1}^{n}{\left(P_{i}-Y_{i}\right)}^{2}.$$

The *R* ^2^ score is the coefficient of determination and is used to measure how well the regression model fits data. It shows the percentage of variance in the dependent variable explained by the independent variables. In other words, it is an indicator of how well a regression model is fitting the data that it is estimating. An *R* ^2^ score if it scores 1, the model perfectly predicts the dependent variable, and if it is 0, the model does not explain any of the variability in the dependent variable, as represented in Equation (6),

(6) $$R^{2}=1-\frac{{\text{SS}}_{R}}{{\text{SS}}_{M}},$$

where ${\text{SS}}_{R}$ is sum of squared error by the regression line ${\text{SS}}_{R}$ for the sum of squared error by the mean line, where that mean will give a comparison of the regression line with respect to the mean line, as shown in Equation (6). So, the *R* ^2^ score will vary from 0 to 1. It will be zero when the sum of squared error by the mean line becomes equal to the sum of squared error by the regression line, and 1 when the sum of squared error by regression line is zero in Equation (7),

(7) $$\text{CI}=P{\left(1+\frac{r}{n}\right)}^{nt},$$

where compound interest is the interest on interest and refers to the interest that is either earned or charged on the principal amount plus interest, and this is not solely on the initial principal. It is basically “interest on interest,” and it can drastically increase savings or debt liabilities over time. More than one time per year gets additional interest paid based on your interest rate. A variety of compounding is available: annually, semi‐annually, quarterly, monthly, and even daily. Unlike other GCN‐based methods, DeepDTA uses a CNN for predicting drug−target interaction, utilizing sequence‐based rather than graph‐based representation for feature extraction. Training consisted of 1000 epochs and a mini‐batch size of 32 for weight adjustments in the network. For training, we applied the Adam optimization method with a fixed learning rate set to 0.0005. We used Keras’ embedding layer to convert characters into 128‐dimensional dense vectors.

### 5.4 Random forest

**FIGURE 9:**
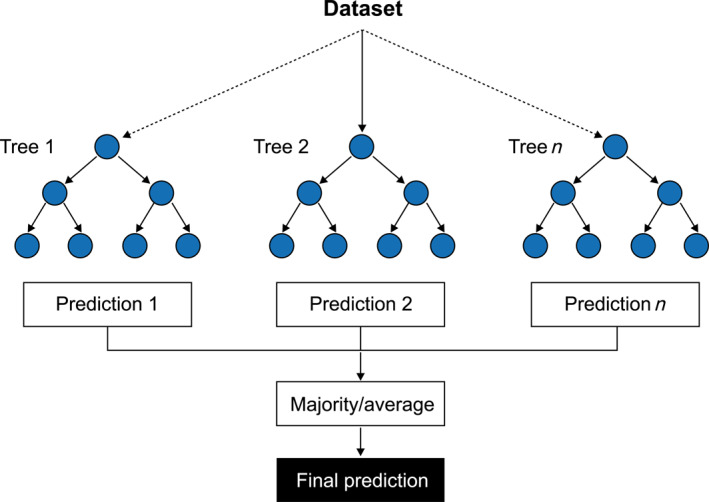
Random forest architecture.

The random forest (RF) model (Figure 9) is a non‐parametric ensemble learning technique that integrates several decision trees [54]. It is frequently employed for regression, classification, and feature selection tasks because of its swift processing speed, high accuracy, and efficacy in parameter optimization. This indicates that for every tree, the algorithm generates sample sets of the data with replacement and trains each tree on these distinct bootstrap datasets [55].

This indicates that from the complete training dataset, 2/3 will serve as in‐bag samples for training, whereas the remaining 1/3 will function as out‐of‐bag samples for validation. The bootstrap approach effectively indicates the predictive accuracy of the model by diminishing prediction variance and so reducing model error [50, 56, 57]. The literature on RF related to GPM is now considerable, and interested readers are directed to these papers for comprehensive information on RF [58].

### 5.5 XGBoost

**FIGURE 10:**
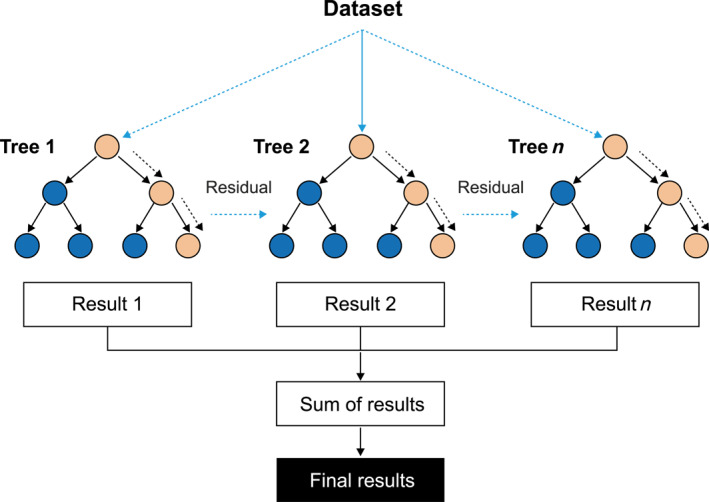
XGBoost architecture.

XGBoost is a scalable supervised gradient boosting method that is extensively utilized for attaining superior performance in diverse learning problems (Figure 10). It enhances computing efficiency by aggregating a cohort of weak tree learners in parallel to create a more robust learner, exceeding the conventional boosting approach [59]. XGBoost utilizes a second‐order Taylor expansion, improving accuracy and enabling customized loss functions through gradient descent. It incorporates tree model complexity into the regularization term to mitigate overfitting, hence enhancing generalization performance [58].

Shrinkage is utilized to diminish the impact of individual trees, hence mitigating bias, whereas column sampling incorporates randomization to lower variation relative to traditional boosting techniques [60]. The primary objective of XGBoost is to minimize the subsequent objective function.

(8) $$L^{\left(t\right)}=\sum_{i=1}^{n}l\left(y_{i}-{\hat{y_{i}}}^{\left(t-1\right)}+f_{t}\left(x_{i}\right)\right)+\Omega \left(f_{t}\right),$$

where *l* denotes the loss function aimed at minimizing errors, and *t* represents the number of iterations; Ω(*f*_*t*_) is the supplementary regularization term that aids in regulating model complexity and mitigating overfitting. *y*_*i*_ represents the actual observed value, whereas $\hat{y_{i}}$ denotes the anticipated value derived using an Equation (8).

(9) $$f_{k}=\gamma T+\frac{1}{2}\lambda |\left|w^{2}\right||,$$

where *γ* and *λ* are the regularization parameters, *ω* represents the scores of each leaf, and *T* is the total number of leaves in the tree (9). For comprehensive descriptions and elucidations of XGBoost, as described by Chen and Guestrin [59].

### 5.6 Experiment workflow

**FIGURE 11:**
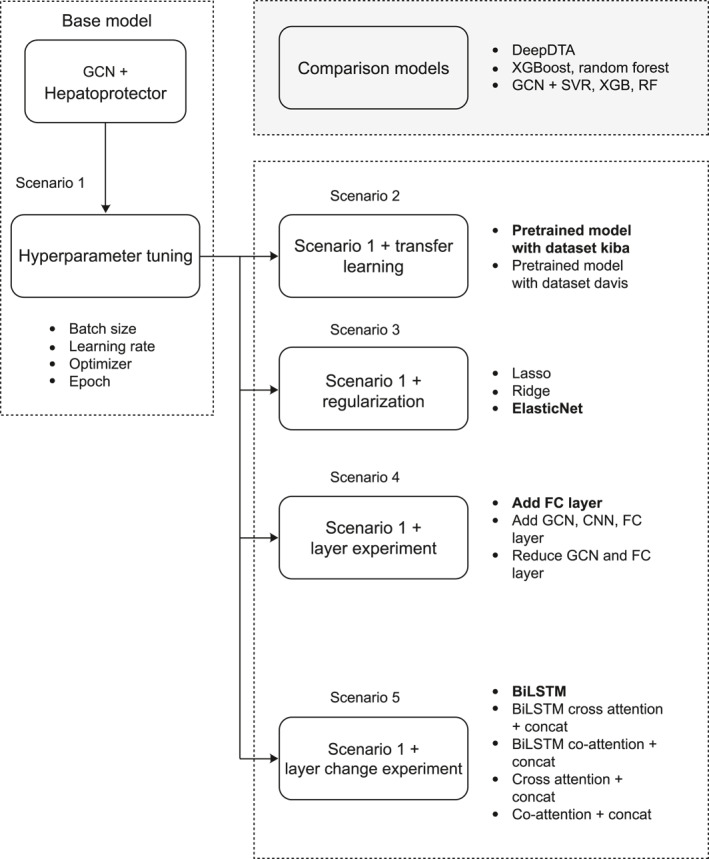
Experiment workflow. Starting from the scratch model, followed by hyperparameter tuning. To get good model performance values, transfer learning, regularization, layer experiments, and layer change experiments and scenarios were added.

The workflow of Figure 11 presents the experimental data regarding the optimization process of a graph convolutional neural network (GCN) applied to hepatoprotector studies. This starts with scenario 1—Hyperparameter tuning (tuning batch size, learning rate, optimizer, and epoch of the model). After that initial step, there are a few experimental directions that branch out from this foundation in order to try different techniques on the model. Scenario 2 includes transfer learning with datasets such as Davis and KIBA to enhance the model’s prediction ability. Scenario 3—Incorporation of regularization methods (Lasso, Ridge, and ElasticNet) for improved generalization and prevention of overfitting. These, among other scenarios, serve as key innovations in the evaluation process to ensure a comprehensive assessment of various approaches to enhance the robustness and accuracy of the model.

Consequently, scenario 4 assesses layer adjustments, as having added or removed FC, GCN, and CNN layers may affect the performance. Finally, in scenario 5 we explore layer change experiments, where we introduce bidirectional LSTM (BiLSTM), cross‐attention mechanism, and concatenation‐based strategies to improve both the feature extraction and sequence learning process. In response to the need for interpretation, BiGraph‐DTA is developed and emerges as the model with the best performance from the evaluation process. The BiGraph‐DTA for predicting interaction of hepatoprotectors with their targets is the main novelty achieved in this work based on a state‐of‐the‐art DL architecture. A concatenation of the feature extraction layers is followed by a FC layer to predict hepatoprotective activity through regression. The model was implemented and tested on a system with the following specifications: Processor—Intel(R) Xeon(R) W‐2223 CPU @ 3.60GHz 3.60 GHz, installed RAM—64.0 GB, system type—64‐bit operating system, x64‐based processor, edition—Windows 11 Pro for workstations, and GPU—RTX A2000.

## AUTHOR CONTRIBUTIONS

**Arief Sartono:** Conceptualization; data curation; formal analysis; investigation; methodology; resources; software; validation; visualization; writing—original draft; writing—review and editing. **Bambang Riyanto Trilaksono:** Data curation; formal analysis; investigation; supervision; writing—review. **Sophi Damayanti:** Data curation; resources; supervision; writing—review. **Anto Satriyo Nugroho:** Data curation; formal analysis; resources; writing—review. **Firdayani Firdayani:** Data curation; investigation; visualization; writing—review.

## CONFLICT OF INTEREST STATEMENT

The authors declare no conflicts of interest.

## ETHICS STATEMENT

This article does not contain any studies with human or animal materials performed by any of the authors.

## Data Availability

The hepatoprotector dataset can be accessed at the ChEMBL website.
